# Evidence of 11-Hydroxy-hexahydrocannabinol and 11-Nor-9-carboxy-hexahydrocannabinol as Novel Human Metabolites of Δ^9^-Tetrahydrocannabinol

**DOI:** 10.3390/metabo13121169

**Published:** 2023-11-23

**Authors:** Christian Falck Jørgensen, Brian Schou Rasmussen, Kristian Linnet, Ragnar Thomsen

**Affiliations:** Section of Forensic Chemistry, Department of Forensic Medicine, Faculty of Health and Medical Sciences, University of Copenhagen, Frederik V’s vej 11, DK-2100 Copenhagen, Denmarkkristian.linnet@sund.ku.dk (K.L.); ragnar.thomsen@sund.ku.dk (R.T.)

**Keywords:** tetrahydrocannabinol, hexahydrocannabinol, 11-nor-9-carboxy-hexahydrocannabinol, 11-hydroxy-hexahydrocannabinol, human liver microsomes, metabolism

## Abstract

(−)-*trans*-Δ^9^-tetrahydrocannabinol (Δ^9^-THC) is the primary psychoactive compound in the Cannabis sativa plant. Δ^9^-THC undergoes extensive metabolism, with the main human phase I metabolites being 11-hydroxy-tetrahydrocannabinol (11-OH-THC) and 11-nor-9-carboxy-tetrahydrocannabinol (THC-COOH). Early animal studies have indicated that the 9-10 double bond may be reduced in vivo to yield 11-hydroxy-hexahydrocannabinol (11-OH-HHC) and 11-nor-9-carboxy-hexahydrocannabinol (HHC-COOH). These metabolites have not been confirmed in humans. In this study, we aimed to investigate whether this metabolic transformation occurs in humans. A range of cannabinoids and metabolites, including 11-OH-HHC and HHC-COOH, were measured in whole blood from 308 authentic forensic traffic cases, of which 222 were positive for Δ^9^-THC. HHC-COOH and 11-OH-HHC were detected in 84% and 15% of the Δ^9^-THC positive cases, respectively, and the estimated median concentration of HHC-COOH was 7%, relative to that of THC-COOH. To corroborate the in vivo findings, Δ^9^-THC and its metabolites 11-OH-THC and THC-COOH were incubated with pooled human liver microsomes. HHC-COOH was detected in both the Δ^9^-THC and 11-OH-THC incubations, while 11-OH-HHC was only detectable in the 11-OH-THC incubation. Hexahydrocannabinol was not detected in any of the incubations, indicating that it is 11-OH-THC or the corresponding aldehyde that undergoes double bond reduction with subsequent oxidation of the aliphatic alcohol to HHC-COOH. In summary, the presented data provide the first evidence of HHC-COOH and 11-OH-HHC being human phase I metabolites of Δ^9^-THC. These findings have implications for interpretation of analytical results from subjects exposed to Δ^9^-THC or HHC.

## 1. Introduction

**Figure 1:**
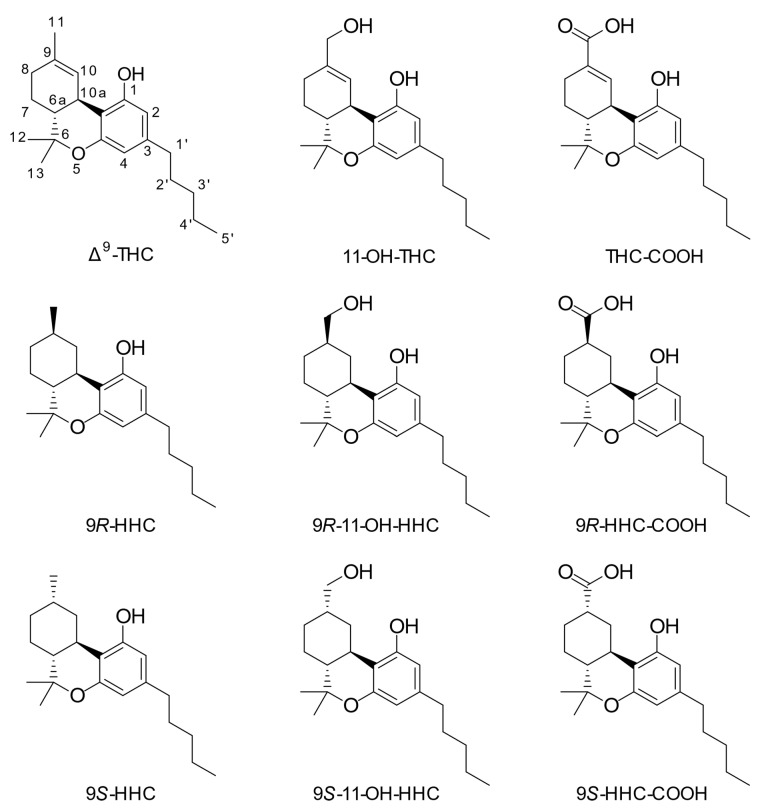
Chemical structures of investigated compounds. The used numbering system is shown for Δ^9^-tetrahydrocannabinol.

(−)-*trans*-Δ^9^-tetrahydrocannabinol (Δ^9^-THC, Figure 1) is the primary psychoactive compound in the Cannabis sativa plant. The metabolism of Δ^9^-THC has been studied in several species, including humans, since the 1970s, and the compound has been demonstrated to undergo extensive phase I and II metabolism [1]. Known phase I reactions include both aliphatic and allylic hydroxylations, with further oxidation to carbonyl compounds and carboxylic acids. Several metabolites incorporating multiple phase I reactions are known to exist, including di- and trihydroxyl compounds, di-acids and hydroxy-acids [2]. Dealkylations and dehydrogenations are known modifications of the pentyl side chain, as well as epoxidation of the main ring alkene [2,3,4,5]. The major phase I metabolites in humans are 11-hydroxy-tetrahydrocannabinol (11-OH-THC) and 11-nor-9-carboxy-tetrahydrocannabinol (THC-COOH) [2,6], with the formation of 11-OH-THC being catalyzed by CYP2C9 and CYP2C19 [3,7]. Other significant hydroxylation sites in humans include the 8-position, which primarily produces the 8β-isomer [6]. Phase II metabolism includes glucuronide, glutathione and fatty acid conjugations [2], with the glucuronide of THC-COOH being the major metabolite excreted in urine [8].

Hexahydrocannabinol (HHC, Figure 1) is a derivative of Δ^9^-THC, wherein the only chemical difference is a reduction of the Δ^9^-double bond, leading to an additional stereocenter and two epimers: 9*R*- and 9*S*-HHC. HHC has gained traction in recent years as a legal alternative to Δ^9^-THC due to its alleged similar pharmacological properties and its relative ease of production from cannabidiol (CBD) [9]. The human metabolism of HHC has not been studied in detail, but liver microsomal studies in a series of rodent species have demonstrated broadly similar hydroxylation patterns to Δ^9^-THC, namely with the 11- and 8-positions predominating in most species [10]. A recent study identified human metabolites in urine after consumption of HHC in two individuals [11]. These findings suggest that various hydroxylated metabolites are produced, including hydroxylations at the C-11 and C-8 positions, as well as on the pentyl side chain. Oxidation at the C-11 position seems less predominant for HHC than Δ^9^-THC. Furthermore, Manier et al. [12] studied the metabolism of HHC using plasma from a single human individual, as well as urine and feces from rat and human liver S9 fractions. They found evidence indicating a metabolic pathway from HHC to 11-OH-HHC and HHC-COOH. Their findings were based on high-resolution mass spectra but were not supported by the analysis of reference material for the metabolites.

In 1977, Harvey and coworkers detected metabolites where the 9,10-double bond had been reduced in the livers of mice administered Δ^9^-THC or Δ^8^-tetrahydrocannabinol (Δ^8^-THC) intraperitoneally [13]. The metabolites were identified as the 9*R*- and 9*S*-epimers (also known as the β- and α-epimers, respectively) of 11-nor-9-carboxy-hexahydrocannabinol (HHC-COOH, Figure 1). The 9*R*- and 9*S*-epimers of 11-hydroxyhexahydrocannabinol (11-OH-HHC, Figure 1) were also detected in the mice administered Δ^8^-THC. The same group demonstrated that the livers of mice administered methyl and ethyl homologs of Δ^9^-THC and Δ^8^-THC contained the corresponding 11-nor-9-carboxy metabolites with the reduced double bond [14,15]. Despite these early studies in mice, reduction of the double bond in the 9,10-position of Δ^9^-THC has, to our knowledge, never been demonstrated to occur in humans.

During the inclusion of HHC in the department’s quantitative, liquid chromatography–mass spectrometry-based analysis for cannabinoids in blood, what appeared to be 11-nor-9-carboxy-hexahydrocannabinol was observed in a large number of the Δ^9^-THC-positive cases. This led to the design of the present study, with the aim to examine whether the reduction of the double bond of Δ^9^-THC takes place in humans. This study is based on the analysis of whole blood from forensic traffic cases supported by data from in vitro experiments employing human liver microsomes.

## 2. Materials and Methods

### 2.1. Chemicals and Reagents

(6a*R*,9*R*,10a*R*)-11-hydroxyhexahydrocannabinol, (6a*R*,9*S*,10a*R*)-11-hydroxyhexahydrocannabinol, (6a*R*,9*R*,10a*R*)-11-nor-9-carboxy-hexahydrocannabinol and (6a*R*,9*S*,10a*R*)-11-nor-9-carboxy-hexahydrocannabinol were from Cayman Chemical (Ann Arbor, MI, USA). (±)-*trans*-11-hydroxy-Δ^9^-tetrahydrocannabinol, (−)-*trans*-Δ^9^-tetrahydrocannabinol and (−)-*trans*-11-nor-9-carboxy-Δ^9^-tetrahydrocannabinol were from Lipomed (Arlesheim, Switzerland). (±)-*trans*-11-hydroxy-Δ^9^-tetrahydrocannabinol, (−)-*trans*-11-nor-9-carboxy-Δ^9^-tetrahydrocannabinol, (−)-*trans*-Δ^8^-tetrahydrocannabinol, (−)-*trans*-Δ^9^-tetrahydrocannabinol-d3, (±)-*trans*-11-hydroxy-Δ^9^-tetrahydrocannabinol-d3 and (±)-*trans*-11-nor-9-carboxy-Δ^9^-tetrahydrocannabinol-d3 were from Cerilliant (Round Rock, TX, USA). (−)-*trans*-Δ^9^-tetrahydrocannabinol and (−)-*trans*-Δ^8^-tetrahydrocannabinol were from LGC (Teddington, UK). Pooled human liver microsomes were from Sigma-Aldrich (St. Louis, MO, USA) and came from 28 individual mixed-gender donors. NADPH regenerating system solutions A and B were from Corning Inc. (Corning, NY, USA).

Other solvents and chemicals were commercially available and of analytical or liquid chromatography–mass spectrometry (LC-MS) grade.

### 2.2. Sample Preparation

Whole blood samples were prepared as described by Andersen et al. [16]. Briefly, the samples were prepared on a fully automated robotic setup. First, the samples were treated by protein precipitation with 15% methanol in acetonitrile, in a 3:1 ratio, followed by solid-phase extraction of the diluted supernatant on Strata X-C columns (Phenomenex, Torrance, CA, USA). The eluents were evaporated to dryness under nitrogen and reconstituted in 8:2 methanol:2 mM ammonium acetate.

### 2.3. Liquid Chromatography–Mass Spectrometry Analysis

Analysis was performed on an ACQUITY UPLC I-class system coupled with a Xevo TQ-S tandem mass spectrometer (both from Waters, Milford, MA, USA), operated in positive-ion electrospray mode and using multiple-reaction monitoring. Chromatographic separation was performed on a Kinetex EVO C18 2.6 µm column (100 mm × 2.1 mm) (Phenomenex, Torrance, CA, USA), which was maintained at a temperature of 45 °C. The mobile phase was composed of solvents A (1 mM ammonium formate with 0.1% formic acid) and B (1:1 methanol:acetonitrile with 0.1% formic acid), delivered at a flow rate of 0.8 mL/min. The gradient had a total run-time of 6.8 min, starting at 60% B, increasing to 80% B over 5 min, increasing to 100% over 0.5 min and isocratic for 0.5 min before re-equilibration for 0.8 min. The injection volume was 10 µL. The method included mass transitions for Δ^9^-THC, HHC, 11-OH-THC, THC-COOH, 11-OH-HHC and HHC-COOH, as well as deuterated analogs Δ^9^-THC-d3, 11-OH-THC-d3 and THC-COOH-d3. The employed mass transitions with experimental settings are provided in Table 1. Figure S1 shows chromatograms from injection of a standard solution containing all analytes.

Identification criteria were retention times +/− 0.02 min and quantifier/qualifier ion transition ratio +/− 20% relative to a standard solution. A peak area cutoff of 1000 for Δ^9^-THC, 11-OH-THC, THC-COOH and HHC-COOH and 2000 for HHC and 11-OH-HHC was employed. In addition to Δ^9^-THC, all samples were screened for the presence of Δ^8^-THC and samples containing significant amounts of Δ^8^-THC or Δ^8^-THC-COOH were excluded. For each analyte in a sample, a response factor was calculated by dividing the analyte peak area by the internal standard peak area. The response factors were used for all further data processing. Internal standards utilized are shown in Table S1. Samples where the recovery of the deuterated internal standards was lower than 50% compared to the calibrators were excluded from the analysis.

For the forensic traffic cases, a six-point calibration curve with concentrations of 0.53, 1.325, 5.3, 13.25, 26.5 and 53 ng/mL was used to quantify the following compounds: Δ^9^-THC, 11-OH-THC and THC-COOH. The lower limit of quantification (LLOQ) for Δ^9^-THC and 11-OH-THC was 0.53 ng/mL and 1.06 ng/mL for THC-COOH. 

Reference materials for HHC, HHC-COOH and 11-OH-HHC were not available at the time of analysis; therefore, concentrations were estimated using another compound’s calibration curve and a correction factor for the response difference between the analytes. The calibration curve used for each analyte and correction factors for responses can be found in Table S1. The correction factors were calculated as the ratio between response factors for pairs of analytes. The response factors were determined by analysis of a mixture of all analytes at a concentration of 2.5 ng/mL, which was analyzed on six different days. The LLOQs of Δ^9^-THC, 11-OH-THC and THC-COOH were used for the rest of the analytes, as specified in Table S1.

### 2.4. Incubation in Human Liver Microsomes

In total, 5 µM of Δ^9^-THC, 11-OH-THC or THC-COOH were incubated in phosphate buffer at a total volume of 250 μL with 1 mg protein/mL pooled human liver microsomes, 1.3 mM NADP+, 3.3 mM glucose-6-phosphate, 3.3 mM MgCl_2_, 0.4 U/mL glucose-6-phosphate dehydrogenase and 0.05 mM sodium citrate. The temperature was kept at 37 °C during the incubation. The reaction was initiated after 1 min pre-incubation by the addition of NADPH regenerating system solution B. At timepoints 20, 120 and 180 min, 20 μL aliquots of the incubation were transferred to 50 μL ice-cold methanol, containing an internal standard mixture and subsequentially diluted with 30 µL water. Incubations without NADPH regenerating system (solutions A and B) served as negative controls. All incubations were made in duplicate.

### 2.5. Forensic Traffic Cases

Anonymized whole blood samples from authentic forensic traffic cases received from the capital region of Denmark entered the study. In total, 308 consecutive blood samples collected in the months of June and July 2023 were subjected to the described analytical method.

### 2.6. Data Analysis

Data were acquired with MassLynx 4.2 (Waters, Milford, MA, USA), while peak integration was performed with TargetLynx XS 4.2 (Waters). The processed data were converted to an XML file and imported into Python 3.10.5, where the rest of the data analysis was performed. The following Python packages were utilized: Pandas [17], Plotly 5.10.0 [18], NumPy 1.23.1 [19] and SciPy 1.9.0.3 [20].

## 3. Results

### 3.1. Traffic Cases

**Figure 2:**
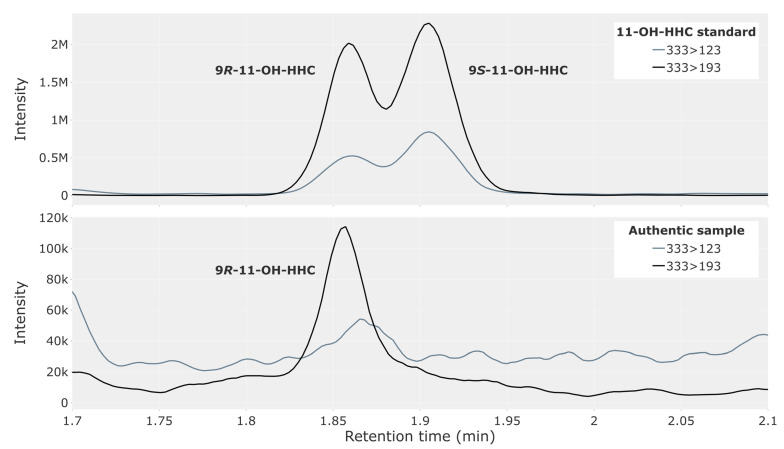
Chromatogram showing 11-hydroxy-hexahydrocannabinol (11-OH-HHC) in a reference solution containing both the 9*R*- and 9*S*-epimer at 2.5 ng/mL (**top**) and in whole blood from a representative authentic forensic traffic case (**bottom**).

The 9*R*- and 9*S*-epimers of 11-OH-HHC were not baseline-separated with the present method; however, a clear indication of the two isomers is evident from the top chromatogram in Figure 2. The bottom chromatogram of Figure 2 shows an analysis of whole blood from a representative traffic case with an abundant peak for the 9*R*-epimer of 11-OH-HHC.

**Figure 3:**
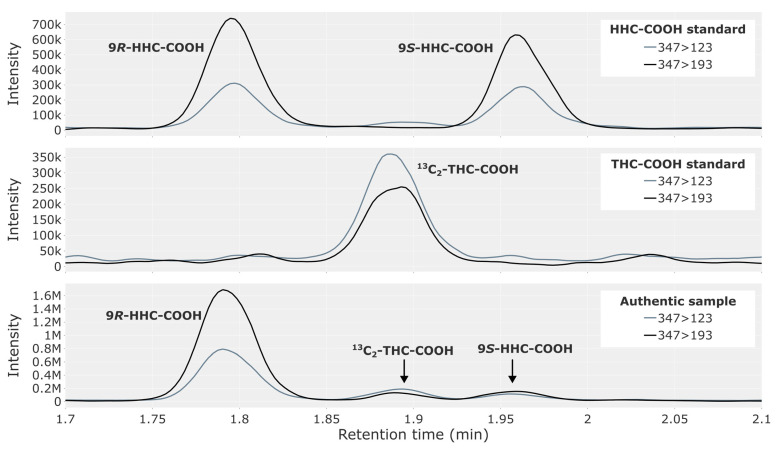
Chromatogram showing HHC-COOH in a reference solution containing both the 9*R*- and 9*S*-epimer at 2.5 ng/mL (**top**), ^13^C_2_-THC-COOH from a THC-COOH reference solution (**middle**) and a whole blood sample from a representative authentic forensic traffic case (**bottom**), where a peak for ^13^C_2_-THC-COOH is visible.

The two epimers of HHC-COOH were completely baseline-separated, with retention times on either side of the THC-COOH peak (Figure 3, top). The representative traffic case (Figure 3, bottom) contained a large peak for 9*R*-HHC-COOH, with only trace amounts of the 9*S*-epimer. High concentrations of THC-COOH produce a chromatographic peak between the two epimers, resulting from THC-COOH containing two carbon-13 isotopes (Figure 3, middle).

Table 2 provides an overview of the results from the traffic cases. Since 11-OH-HHC and HHC-COOH have been proposed as likely human metabolites of HHC [9], the cases were grouped by the presence of Δ^9^-THC and HHC. In total, 308 cases were analyzed. Of them, 222 were positive for Δ^9^-THC (Group A), 10 were positive for both Δ^9^-THC and HHC (Group B), and 76 were negative for both (Group C). There were no cases that were only positive for HHC in the collection period. Of the Group A cases, a majority were positive for 9*R*-HHC-COOH (84%) and 9*S*-HHC-COOH (50%). All cases in Group B were positive for 9*R*-HHC-COOH, while 30% were positive for 9*S*-HHC-COOH. 11-OH-HHC had a lower prevalence than HHC-COOH in the traffic cases, where only the 9*R*-epimer was detected in 15% of the Group A cases. In contrast, the known major metabolites of Δ^9^-THC, 11-OH-THC and THC-COOH were detected in 80% and 92% of the cases in Group A, respectively.

Of the cases negative for THC and HHC (Group C), only two and one case(s) were positive for 9*R*-HHC-COOH and THC-COOH, respectively, while none were positive for the 11-hydroxy metabolites.

In Table 3, results from quantification of Δ^9^-THC, 11-OH-THC and THC-COOH in cases from Group A are shown, as well as estimated concentrations for 9*R*-HHC-COOH and 9*S*-HHC-COOH. Concentrations for 9*R*-11-OH-HHC and 9*S*-11-OH-HHC are not shown due to the low number of positive cases. Median concentrations of Δ^9^-THC, 11-OH-THC and THC-COOH were comparable between Groups A and B. In Group A, the median concentrations of 9*R*-HHC-COOH, THC-COOH and 11-OH-THC were 1.4, 21 and 1.1 ng/mL, respectively. The concentrations of 9*S*-HHC-COOH were noticeably lower compared to those of 9*R*-HHC-COOH, resulting in a median concentration of 0 since half of the cases were negative. For all compounds, the minimum concentration was below the LLOQ.

### 3.2. Incubation with Human Liver Microsomes

**Figure 4:**
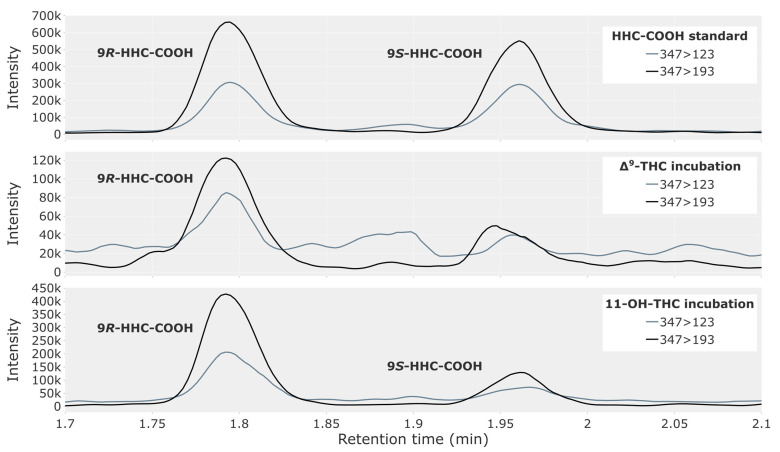
Chromatogram showing the 9*R*- and 9*S*-epimers of HHC-COOH in a reference solution of 2.5 ng/mL (**top**) and in a 180 min incubation of pooled human liver microsomes, with 5 µM of either Δ^9^-THC (**middle**) or 11-OH-THC (**bottom**).

Δ^9^-THC, 11-OH-THC or THC-COOH were incubated with pooled human liver microsomes. Table 4 provides an overview of the incubation results. At the 120 and 180 min timepoints, the Δ^9^-THC and 11-OH-THC incubations produced a significant chromatographic peak for 9*R*-HHC-COOH (Figure 4), which fulfilled the criteria for retention time and ion ratio as specified in the Materials and Methods section. The peak for 9*S*-HHC-COOH was smaller and only confirmed by retention time and ion ratio in the 11-OH-THC incubation. No peaks for HHC-COOH were observed at the 20 min timepoint or in any of the negative controls (Figures S3, S4 and S6). The THC-COOH incubation produced no peaks for HHC-COOH at any timepoints (Figure S6).

It was not possible to detect 11-OH-HHC in the Δ^9^-THC incubation (Figure S2). There was a peak with the correct retention time for the quantifier ion at 120 and 180 min, but the concentration was likely too low to produce a signal for the qualifier ion. Also, the THC-COOH incubation produced no peaks for 11-OH-HHC at any timepoints (Figure S5).

The incubation of Δ^9^-THC formed both 11-OH-THC and THC-COOH (Figures S7 and S9), confirming the known metabolism of the drug and the enzymatic activity of the assay.

Furthermore, the incubation of 11-OH-THC produced THC-COOH at the 120 and 180 min timepoints, as expected (Figure S10). None of the incubations produced either epimer of HHC.

After 180 min, the substrate was depleted in the Δ^9^-THC incubation, approximately 15% substrate remained in the 11-OH-THC incubation, while the substrate amount remained unchanged in the THC-COOH incubation.

Due to the reference material for 11-OH-THC containing 11-OH-HHC as an impurity (Figure S11), there were peaks for both 9*R*- and 9*S*-11-OH-HHC in all 11-OH-THC incubations, including the negative controls. Thus, it was not possible to directly confirm the formation of 11-OH-HHC from 11-OH-THC in the microsomal studies, and for that reason, blank positions are left in Table 4.

## 4. Discussion

The majority of cases in Group A were positive for HHC-COOH, which is highly suggestive of in vivo generation of HHC-COOH from Δ^9^-THC. The known metabolite THC-COOH was detected in 92% of the cases in Group A. The median concentrations of THC-COOH and 9*R*-HHC-COOH were 21 ng/mL and 1.4 ng/mL, respectively. Thus, both THC-COOH and 9*R*-HHC-COOH were detected in most of the cases in Group A where the median concentration of 9R-HHC-COOH was 7% of that of THC-COOH. 9*R*-HHC-COOH thus appears to be a common metabolite of HHC and Δ^9^-THC and may not prove to be a suitable target for establishing a specific intake of HHC.

Unfortunately, since there were no cases only positive for HHC, it was not possible to establish from the present data whether HHC-COOH is also a human metabolite of HHC. The lack of HHC-only cases suggests that HHC users in the Danish population also commonly ingest Δ^9^-THC.

Of the cases in Group A, 11-OH-HHC had a low prevalence. The concentration in most cases was below the LLOQ, while the known metabolite 11-OH-THC was found in most cases. This suggests that 11-OH-HHC is produced in lower amounts than 11-OH-THC in vivo and is efficiently oxidized further to HHC-COOH. This is analogous to 11-OH-THC typically having a lower blood concentration than THC-COOH upon ingestion of Δ^9^-THC [1]. Based on the present data, we cannot rule out further hydroxylation of 11-OH-HHC, leading to di- and tri-hydroxylated metabolites as is known for 11-OH-THC. Only three cases in Group C were positive for any of the metabolites. These few cases are likely a result of sampling taking place long after drug intake, so the parent drug was cleared from circulation.

In the incubation of Δ^9^-THC and 11-OH-THC with human liver microsomes, significant peaks were formed for HHC-COOH. Thus, it appears that human liver microsomes can metabolize Δ^9^-THC and 11-OH-THC to HHC-COOH, preferentially producing the 9*R*-epimer of HHC-COOH. This is consistent with the 9*R*-epimer being more abundant in the traffic cases. Since no significant peaks were found when incubating THC-COOH, the metabolism does not appear to go through THC-COOH as an intermediate.

It should be noted that the available reference material for 11-OH-THC was a mixture of the *trans*-enantiomeric pair. Thus, less than 100% of 11-OH-THC used as substrate in the incubation was of the natural (−)-*trans*-isomer. It is unknown how this affects the affinity of the potentially involved enzyme(s). However, the incubation with Δ^9^-THC containing only the natural (−)-*trans*-isomer showed similar activity to the 11-OH-THC incubation.

**Figure 5:**
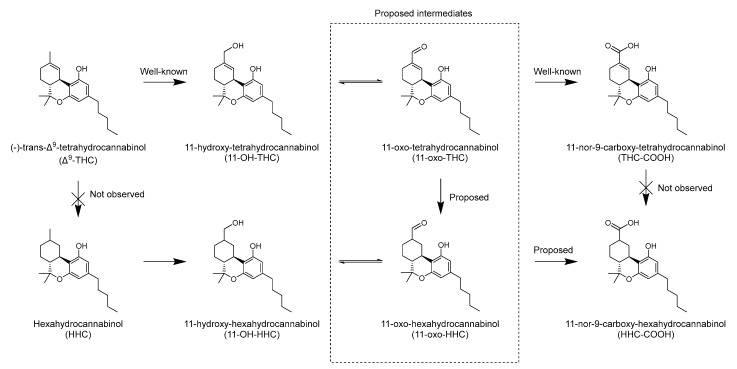
Proposed metabolic pathway for production of 11-hydroxy-hexahydrocannabinol and 11-nor-9-carboxy-hexahydrocannabinol from Δ^9^-tetrahydrocannabinol in humans.

Both the microsomal incubation of Δ^9^-THC and its metabolite 11-OH-THC produced HHC-COOH, while HHC itself was not detected at any timepoint in any of the incubations. The THC-COOH incubation failed to produce HHC-COOH. This indicates that it is not Δ^9^-THC itself but one or more of its oxidative metabolites that undergo the reduction. Since 9*R*-11-OH-HHC was detected in several of the traffic cases, it is reasonable to assume that it is 11-OH-THC or a downstream metabolite, which is reduced with subsequent oxidation to form HHC-COOH. The proposed metabolic pathway from Δ^9^-THC to 11-OH-HHC and HHC-COOH is shown in Figure 5.

A limitation of the current study is that the dose and content of ingested product(s), route of administration and time between drug intake and sampling are unknown. This likely leads to increased variation in the concentration of the measured cannabinoids and metabolites, as well as the ratio between parent drug and metabolites.

The present study does not provide data on which enzyme(s) may catalyze the reduction, apart from the fact that the enzyme(s) is present in the liver microsomal fraction. Additionally, the reduction is stereoselective, producing the 9*R*-epimers in greater amounts compared to the 9*S*-epimers. There are several known examples of enzymatic alkene reduction in humans. Some examples include 5α-reductase, which converts testosterone into the more potent androgen dihydrotestosterone; 7-dehydrocholesterol reductase, which catalyzes the final step in the cholesterol biosynthesis, namely the conversion of 7-dehydrocholesterol to cholesterol; and biliverdin reductase, which converts biliverdin to bilirubin [21,22]. Most examples of alkene reductions in biology require an electron-withdrawing group to activate the alkene, typically through conjugation, like an α,β-unsaturated carbonyl group. Thus, in the case of 11-OH-THC, it is likely that the alkene reduction is preceded by alcohol oxidation to the aldehyde. 11-oxo-tetrahydrocannabinol has been detected in rat liver microsomes and is a likely intermediate in the oxidation of Δ^9^-THC to THC-COOH [23]. Whether any of the above-mentioned enzymes are involved in the reaction needs to be further investigated.

Since there were prominent peaks for HHC-COOH in most of the Δ^9^-THC-positive cases and since the consumption of Δ^9^-THC is so prevalent, one may wonder why this metabolite was not reported in humans previously. This may be due to the plethora of cannabinoids found in the cannabis plant, the many potential isomers, as well as the extensive metabolism of cannabinoids, leading to many compounds present in the blood of cannabis users having the same nominal or exact mass. Another potential explanation is HHC-COOH having the same nominal mass as ^13^C_2_-THC-COOH. Since THC-COOH is abundant in blood from persons having ingested Δ^9^-THC, the HHC-COOH peak may easily be disregarded as ^13^C_2_-THC-COOH, especially in other methods with less chromatographic resolution.

## 5. Conclusions

In the present study, HHC-COOH was observed in whole blood from a majority of the 222 analyzed traffic cases that were positive for Δ^9^-THC, while 11-OH-HHC was observed to a lesser extent. The 9*R*-epimer of both HHC-COOH and 11-OH-HHC predominated. This supports the notion that HHC-COOH and 11-OH-HHC are human phase I metabolites of Δ^9^-THC. These findings were corroborated by in vitro experiments, which demonstrated that human liver microsomes can metabolize Δ^9^-THC to HHC-COOH. The identity of the enzyme(s) involved and the enzymatic mechanism for the reduction of the double bond are still unknown, but the presented in vitro data indicate that the oxidation of Δ^9^-THC to 11-OH-THC precedes the reduction to 11-OH-HHC.

An implication of the presented results for toxicology is that the presence of HHC-COOH or 11-OH-HHC in the blood cannot be used as sole evidence for consumption of HHC when Δ^9^-THC intake cannot be excluded.

## Figures and Tables

**Table 1:** Monitored *m*/*z* transitions with the corresponding cone voltage, collision energy and retention time.

Compound	Monitored *m*/*z* Transitions	Cone Voltage (V)	Collision Energy (eV)	Retention Time (min)
Δ^9^-tetrahydrocannabinol	315.22 > 193.12 * 315.22 > 123.04	42 42	22 32	3.60
9*R*-Hexahydrocannabinol	317.25 > 193.12 * 317.25 > 123.04	20 20	20 40	4.08
9*S*-Hexahydrocannabinol	317.25 > 193.12 * 317.25 > 123.04	20 20	20 40	4.00
9*R*-11-hydroxy-hexahydrocannabinol	333.24 > 193.12 * 333.24 > 123.04	20 20	25 35	1.86
9*S*-11-hydroxy-hexahydrocannabinol	333.24 > 193.12 * 333.24 > 123.04	20 20	25 35	1.91
9*R*-11-nor-carboxy-hexahydrocannabinol	347.22 > 193.12 * 347.22 > 123.04	20 20	25 35	1.80
9*S*-11-nor-carboxy-hexahydrocannabinol	347.22 > 193.12 * 347.22 > 123.04	20 20	25 35	1.97
11-hydroxy-tetrahydrocannabinol	331.22 > 193.12 * 331.22 > 201.20	20 20	29 25	1.71
11-nor-9-carboxy-tetrahydrocannabinol	345.20 > 193.12 * 345.20 > 299.20	42 42	26 20	1.90
Δ^9^-tetrahydrocannabinol-d3	318.22 > 196.12	42	22	3.58
11-hydroxy-tetrahydrocannabinol-d3	334.27 > 196.23	20	29	1.89
11-nor-9-carboxy-tetrahydrocannabinol-d3	348.20 > 196.12	34	20	1.70

* Quantifier transition.

**Table 2:** Number of forensic traffic cases with whole blood samples positive for 9*R*- and 9*S*-epimers of 11-OH-HHC and HHC-COOH, as well as 11-OH-THC and THC-COOH. The cases are grouped according to the presence of Δ^9^-THC and HHC. + and – indicate the presence and absence of a chromatographic peak that fulfilled all criteria for peak identification, respectively.

Group	Δ^9^-THC	HHC	Number of Cases (n = 308)	9*R*-HHC-COOH	9*S*-HHC-COOH	9*R*-11-OH-HHC	9*S*-11-OH-HHC	THC- COOH	11-OH- THC
A	+	−	222	186 (84%)	112 (50%)	33 (15%)	0 (0%)	204 (92%)	178 (80%)
B	+	+	10	10 (100%)	3 (30%)	1 (10%)	1 (10%)	10 (100%)	7 (70%)
C	−	−	76	2 (3%)	0 (0%)	0 (0%)	0 (0%)	1 (1%)	0 (0%)

**Table 3:** Quantitative results from the analysis of whole blood from forensic traffic cases. Only cases positive for Δ^9^-THC and negative for HHC are included, corresponding to Group A in Table 2. The number of cases was 222.

Compound	Median Concentration (ng/mL)	Maximum Concentration (ng/mL)
Δ^9^-THC	2.8	35
11-OH-THC	1.1	14
THC-COOH	21	269
9*R*-HHC-COOH ^a^	1.4	16
9*S*-HHC-COOH ^a^	0	4.4

^a^ Estimated concentrations based on response ratios and calibration curve of THC-COOH.

**Table 4:** Detected metabolites in incubations of Δ^9^-THC, 11-OH-THC or THC-COOH with pooled human liver microsomes. + indicates a chromatographic peak that fulfilled all criteria for peak identification, − indicates no peak and (+) indicates observed chromatographic peaks without fulfilling all criteria for peak identification. Gray areas indicate the incubated compound.

	Δ^9^-THC			11-OH-THC			THC-COOH		
	20 min	120 min	180 min	20 min	120 min	180 min	20 min	120 min	180 min
9*R*-11-OH-HHC ^a^	−	(+)	(+)				−	−	−
9*S*-11-OH-HHC ^a^	−	−	−				−	−	−
9*R*-HHC-COOH	−	+	+	−	+	+	−	−	−
9*S*-HHC-COOH	−	−	−	−	+	+	−	−	−
11-OH-THC	+	+	+				−	−	−
THC-COOH	−	+	+	−	+	+			

^a^ 9*R*-11-OH-HHC and 9*S*-11-OH-HHC are not presented for the 11-OH-THC incubation due to impurities of these compounds in the 11-OH-THC reference material.

## Data Availability

The data presented in this study are available on request from the corresponding author. Data is not publicly available due to privacy.

## Supplementary Materials

The following supporting information can be downloaded at https://www.mdpi.com/article/10.3390/metabo13121169/s1. Figure S1: Chromatograms of a standard solution showing all analytes in the method; Figure S2: Chromatogram showing 9R- and 9S-epimers of 11-hydroxy-hexahydrocannabinol (11-OH-HHC) in incubation of Δ^9^-tetrahydrocannabinol (Δ^9^-THC) with pooled human liver microsomes; Figure S3: Chromatogram showing 9*R*- and 9*S*-epimers of 11-nor-9-carboxy-hexahydrocannabinol (HHC-COOH) in incubation of Δ9-tetrahydrocannabinol (Δ9-THC) with pooled human liver microsomes; Figure S4: Chromatogram showing 9*R*- and 9*S*-epimers of 11-nor-9-carboxy-hexahydrocannabinol (HHC-COOH) in incubation of 11-hydroxy-tetrahydrocannabinol (11-OH-THC) with pooled human liver microsomes; Figure S5: Chromatogram showing 9*R*- and 9*S*-epimers of 11-hydroxy-hexahydrocannabinol (11-OH-HHC) in incubation of 11-nor-9-carboxy-tetrahydrocannabinol (THC-COOH) with pooled human liver microsomes; Figure S6: Chromatogram showing 9*R*- and 9*S*-epimers of 11-nor-9-carboxy-hexahydrocannabinol (HHC-COOH) in incubation of 11-nor-9-carboxy-tetrahydrocannabinol (THC-COOH) with pooled human liver microsomes; Figure S7: Chromatogram showing 11-hydroxy-tetrahydrocannabinol (11-OH-THC) in incubation of Δ^9^-tetrahydrocannabinol (Δ^9^-THC) with pooled human liver microsomes; Figure S8: Chromatogram showing 11-hydroxy-tetrahydrocannabinol (11-OH-THC) in incubation of 11-nor-9-carboxy-tetrahydrocannabinol (THC-COOH) with pooled human liver microsomes; Figure S9: Chromatogram showing 11-nor-9-carboxy-tetrahydrocannabinol (THC-COOH) in incubation of Δ^9^-tetrahydrocannabinol (Δ^9^-THC) with pooled human liver microsomes; Figure S10: Chromatogram showing 11-nor-9-carboxy-tetrahydrocannabinol (THC-COOH) in incubation of 11-hydroxy-tetrahydrocannabinol (11-OH-THC) with pooled human liver microsomes; Figure S11: Chromatogram showing 9*R*- and 9*S*-epimers of 11-hydroxy-hexahydrocannabinol (11-OH-HHC) and 11-hydroxy-tetrahydrocannabinol (11-OH-THC) in a standard solution containing 11-OH-THC; Table S1: Parameters used for quantification of compounds.
