# Supplementary material for: Evidence of 11-Hydroxy-hexahydrocannabinol and 11-Nor-9-carboxy-hexahydrocannabinol as Novel Human Metabolites of Δ9-Tetrahydrocannabinol
Source: Metabolites. 2023 Nov 23;13(12):1169. doi: 10.3390/metabo13121169 (PMC10744638; doi:10.3390/metabo13121169)

Supporting Information

Evidence of 11-hydroxy-hexahydrocannabinol and 11-nor-9-carboxy-hexahydrocannabinol as novel human metabolites of Δ^9-tetrahydrocannabinol

Table S1. Parameters used for quantification of compounds. Correction factors were used to correct for difference in response factor when calibration was not based on the same compound. The correction factors are the ratio between response, at equal concentration, of the correction factor pair.

**Compound**	**Internal standard**	**Calibration curve**	**Correction factor pair**	**Correction factor**	**Lower limit of quantification (LLOQ)**
Δ^9-THC	Δ^9-THC-d3	Δ^9-THC	-	-	0.53 ng/ml
9*R*-HHC	Δ^9-THC-d3	Δ^9-THC	Δ^9-THC/9*R*-HHC	0.62	Δ^9-THC
9*S*- HHC	Δ^9-THC-d3	Δ^9-THC	Δ^9-THC/9*S*-HHC	0.61	Δ^9-THC
11-OH-THC	11-OH-THC-d3	11-OH-THC	-	-	0.53 ng/ml
9*R*-11-OH-HHC	11-OH-THC-d3	11-OH-THC	11-OH-THC/9*R*-11-OH-HHC	0.15	11-OH-THC
9*S*-11-OH-HHC	11-OH-THC-d3	11-OH-THC	11-OH-THC/9*S*-11-OH-HHC	0.11	11-OH-THC
THC-COOH	THC-COOH-d3	THC-COOH	-	-	1.06 ng/ml
9*R*-HHC-COOH	THC-COOH-d3	THC-COOH	THC-COOH/9*R*-HHC-COOH	0.35	THC-COOH
9*S*-HHC-COOH	THC-COOH-d3	THC-COOH	THC-COOH/9*S*-HHC-COOH	0.42	THC-COOH

Figure S1. Chromatograms of a standard solution showing all analytes in the method. Mass transitions: CBN 311>223, CBD 315>193, THC 315>193, HHC 317>193, 11-OH-THC 331>193, 11-OH-HHC 333>193, THC-COOH 345>193 and HHC-COOH 347>193.

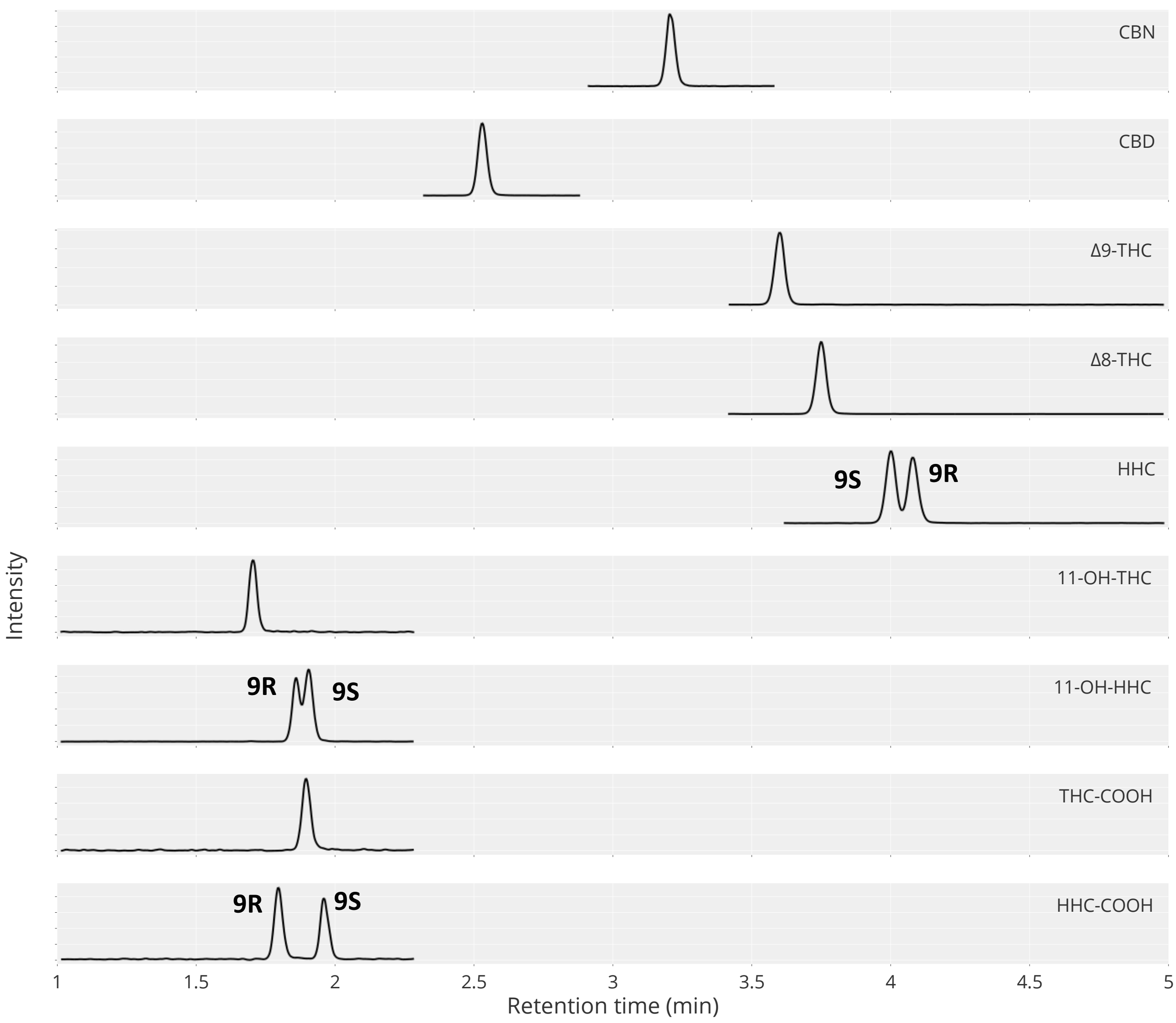

Figure S2. Chromatogram showing 9R- and 9S-epimers of 11-hydroxy-hexahydrocannabinol (11-OH-HHC) in incubation of Δ^9-tetrahydrocannabinol (Δ^9-THC) with pooled human liver microsomes for 20, 120 and 180 minutes. Negative controls are incubations without the NADPH regenerating system. Mass transition: 333>193.

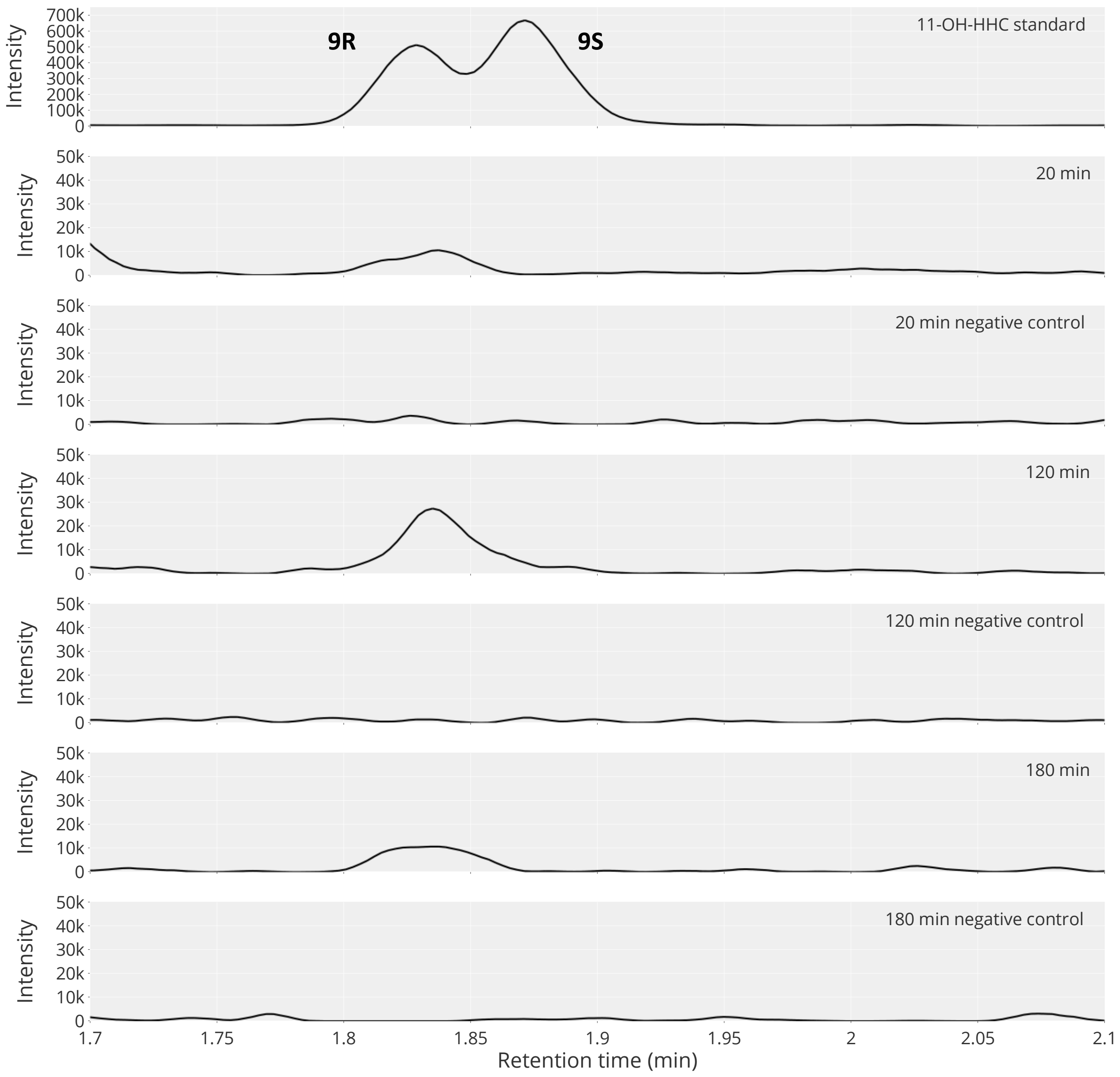

Figure S3. Chromatogram showing 9R- and 9S-epimers of of 11-nor-9-carboxy-hexahydrocannabinol (HHC-COOH) in incubation of Δ[9]-tetrahydrocannabinol (Δ[9]-THC) with pooled human liver microsomes for 20, 120 and 180 minutes. Negative controls are incubations without the NADPH regenerating system. Mass transition: 347>193.

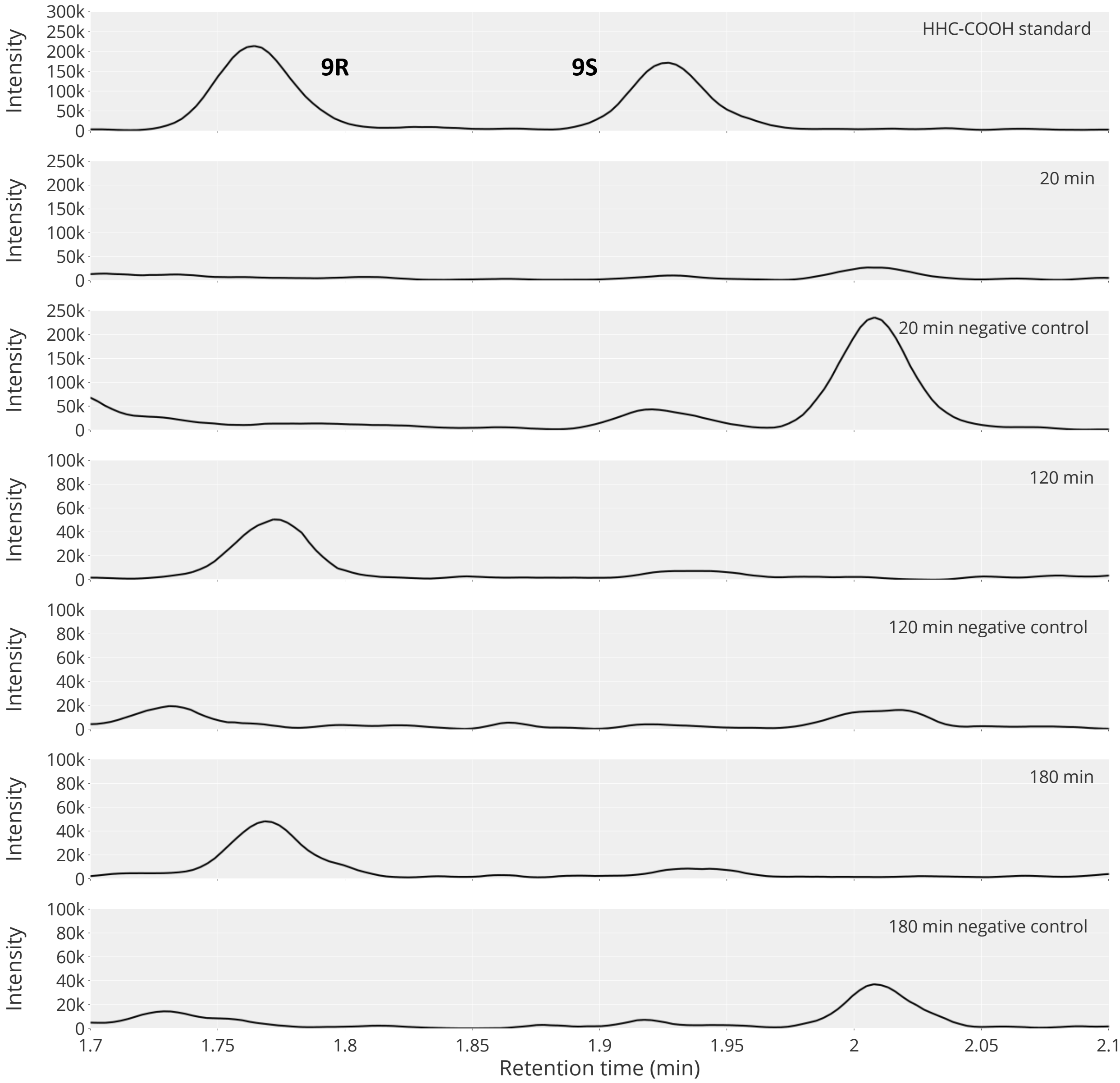

Figure S4. Chromatogram showing 9R- and 9S-epimers of 11-nor-9-carboxy-hexahydrocannabinol (HHC-COOH) in incubation of 11-hydroxy-tetrahydrocannabinol (11-OH-THC) with pooled human liver microsomes for 20, 120 and 180 minutes. Negative controls are incubations without the NADPH regenerating system. Mass transition: 347>193.

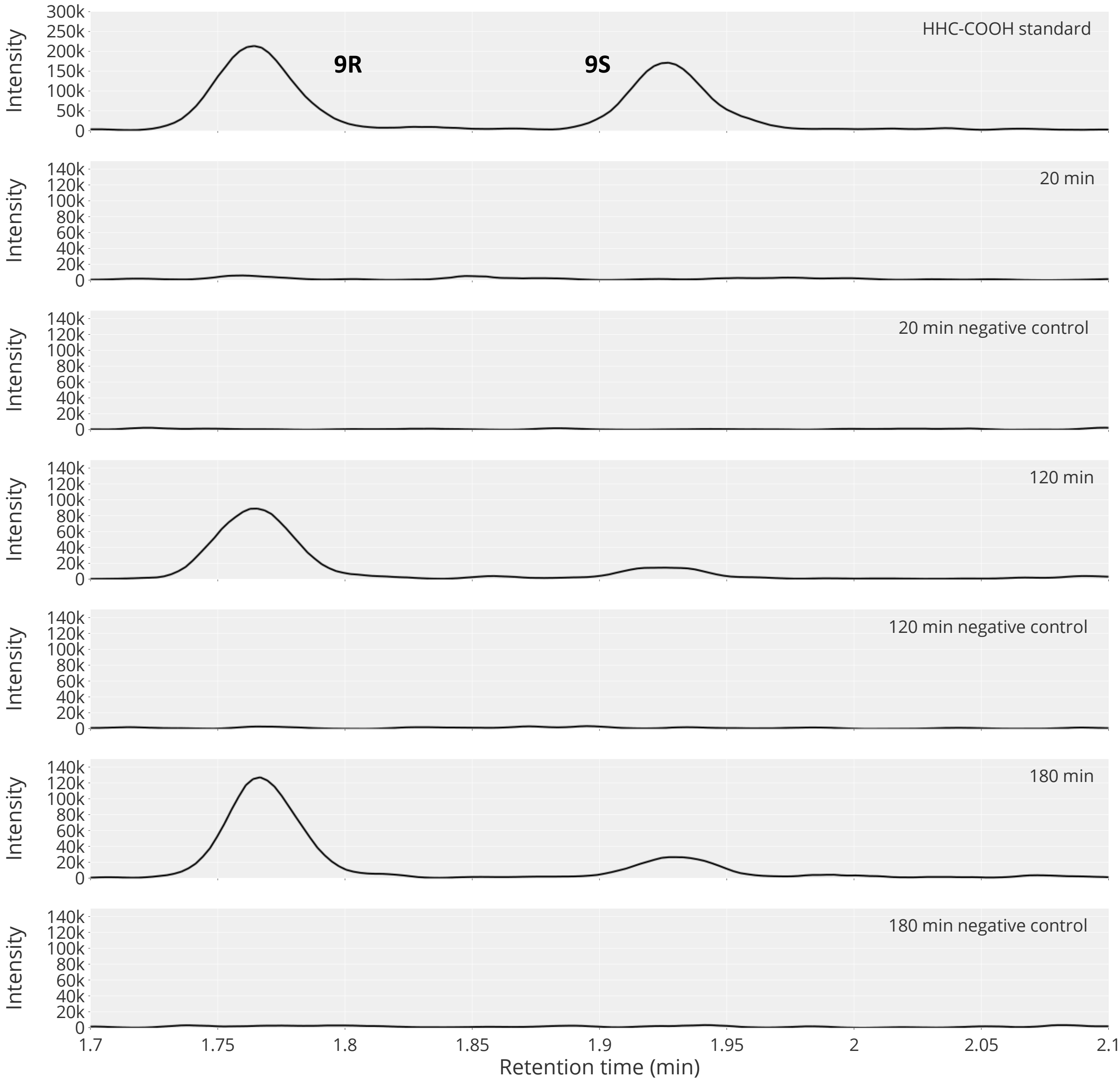

Figure S5. Chromatogram showing 9R- and 9S-epimers of 11-hydroxy-hexahydrocannabinol (11-OH-HHC) in incubation of 11-nor-9-carboxy-tetrahydrocannabinol (THC-COOH) with pooled human liver microsomes for 20, 120 and 180 minutes. Negative controls are incubations without the NADPH regenerating system. Mass transition: 333>193.

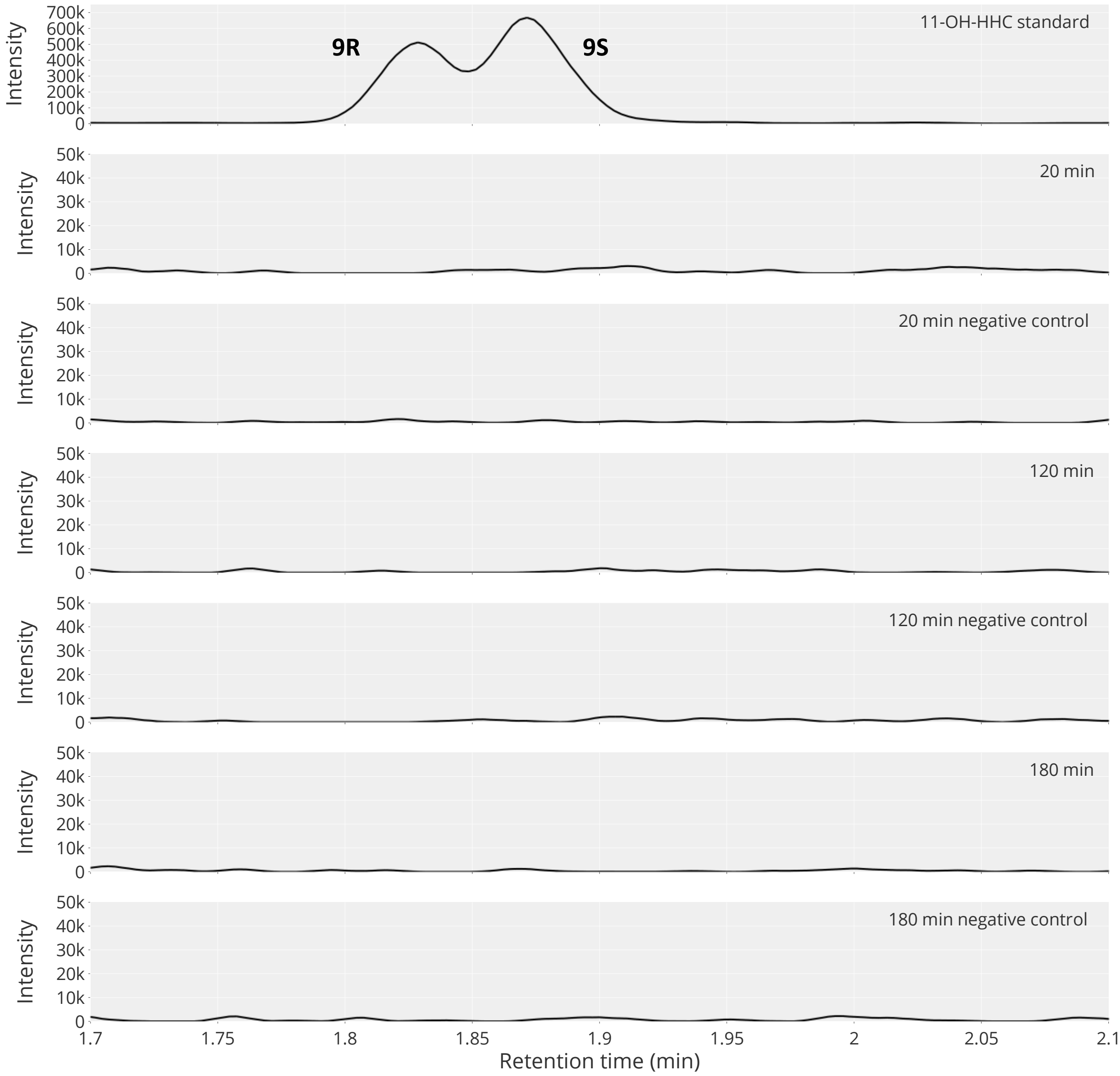

Figure S6. Chromatogram showing 9R- and 9S-epimers of 11-nor-9-carboxy-hexahydrocannabinol (HHC-COOH) in incubation of 11-nor-9-carboxy-tetrahydrocannabinol (THC-COOH) with pooled human liver microsomes for 20, 120 and 180 minutes. Negative controls are incubations without the NADPH regenerating system. Mass transition: 347>193.

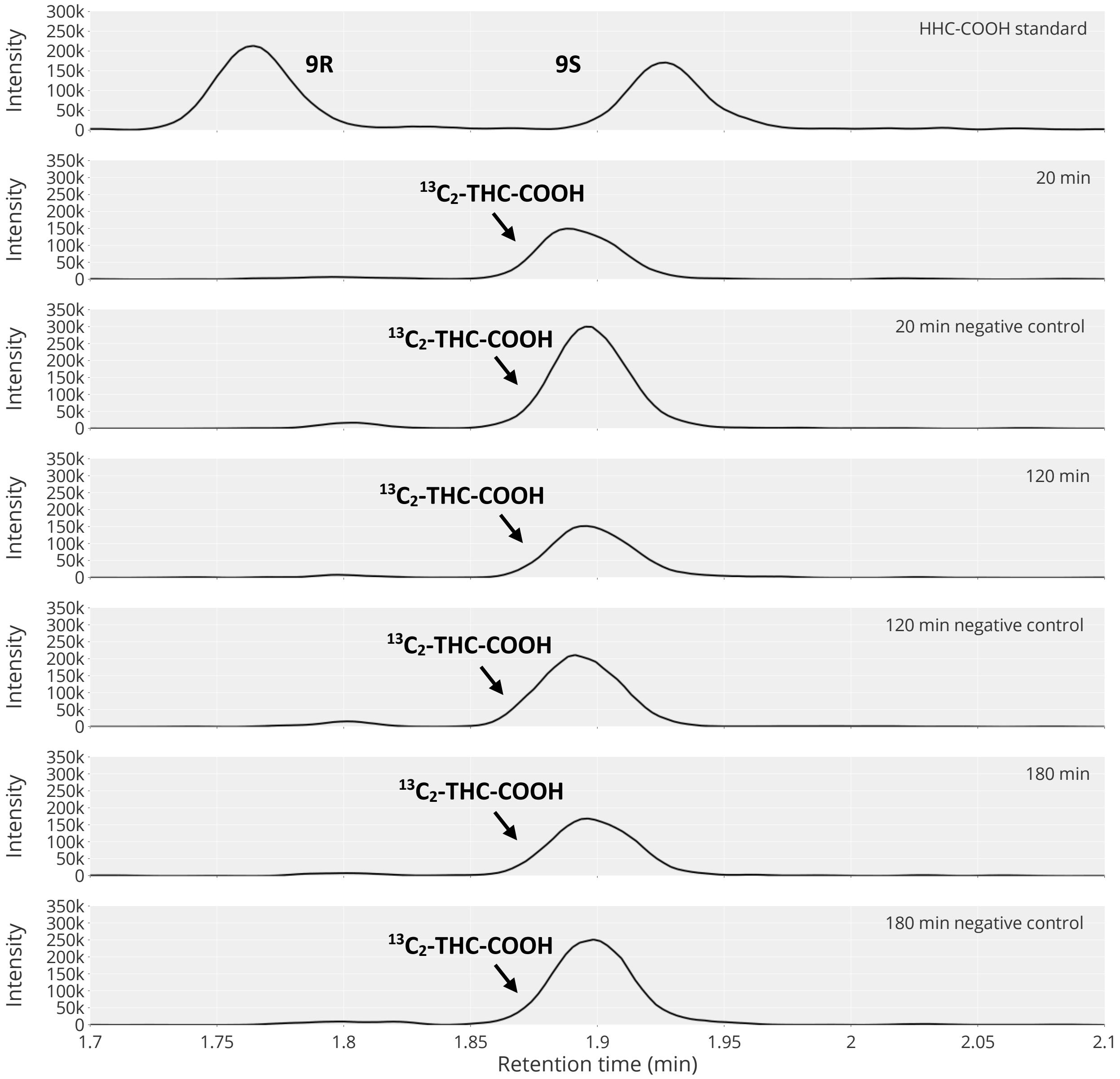

Figure S7. Chromatogram showing 11-hydroxy-tetrahydrocannabinol (11-OH-THC) in incubation of Δ[9]-tetrahydrocannabinol (Δ[9]-THC) with pooled human liver microsomes for 20, 120 and 180 minutes. Negative controls are incubations without the NADPH regenerating system. Mass transition: 331>193.

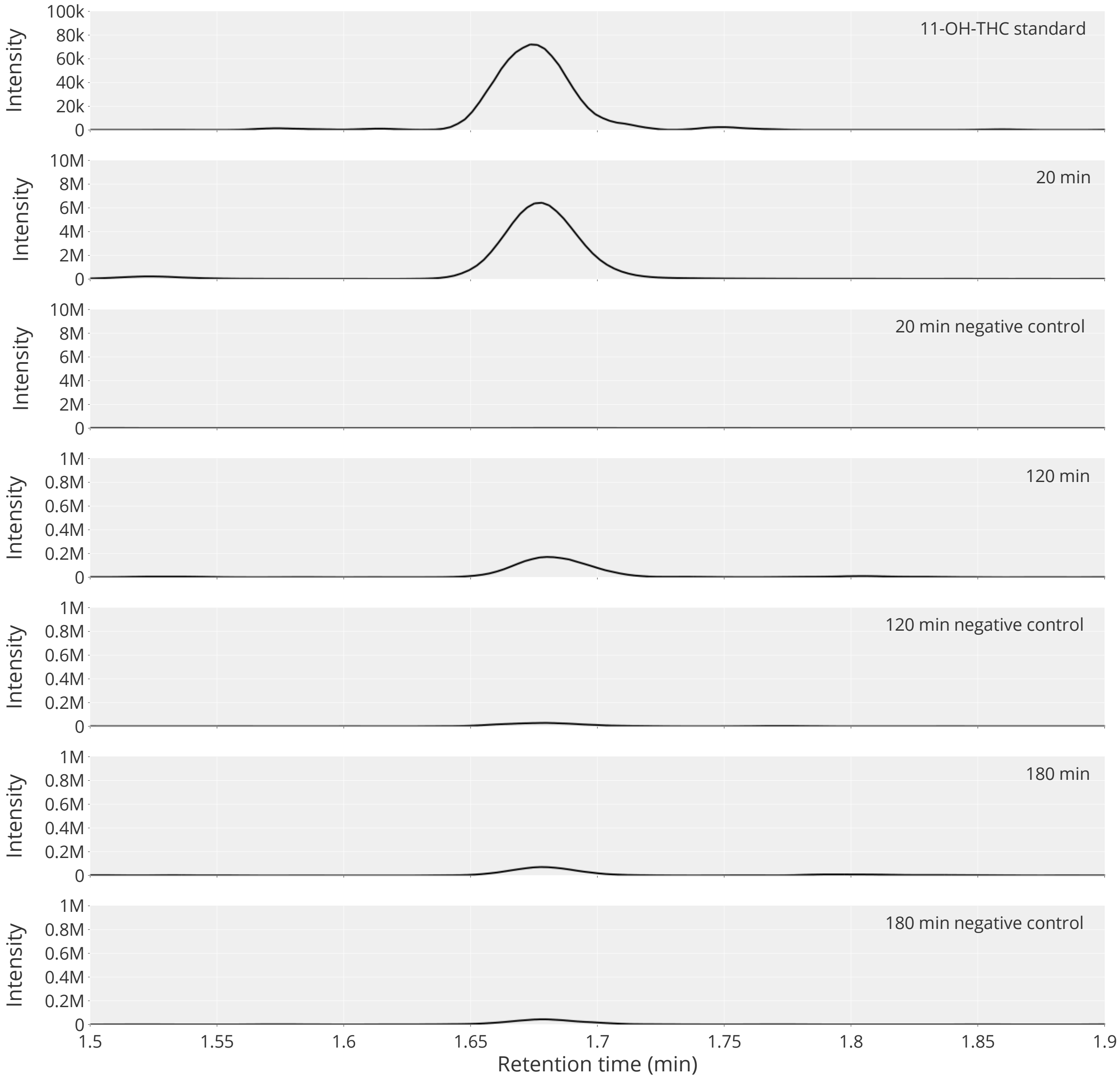

Figure S8. Chromatogram showing 11-hydroxy-tetrahydrocannabinol (11-OH-THC) in incubation of 11-nor-9-carboxy-tetrahydrocannabinol (THC-COOH) with pooled human liver microsomes for 20, 120 and 180 minutes. Negative controls are incubations without the NADPH regenerating system. Mass transition: 331>193.

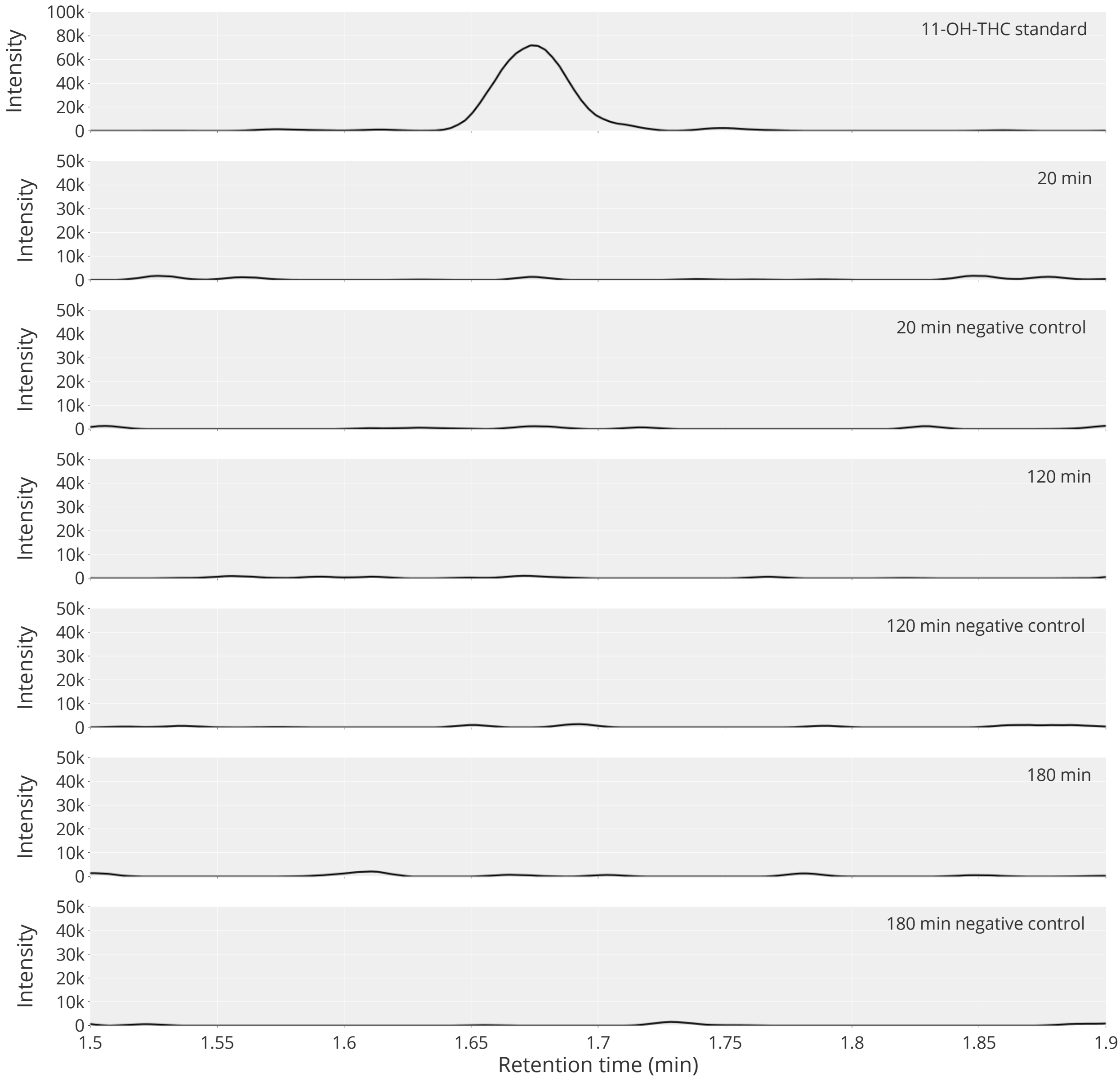

Figure S9. Chromatogram showing 11-nor-9-carboxy-tetrahydrocannabinol (THC-COOH) in incubation of Δ^9-tetrahydrocannabinol (Δ^9-THC) with pooled human liver microsomes for 20, 120 and 180 minutes. Negative controls are incubations without the NADPH regenerating system. Mass transition: 345>193.

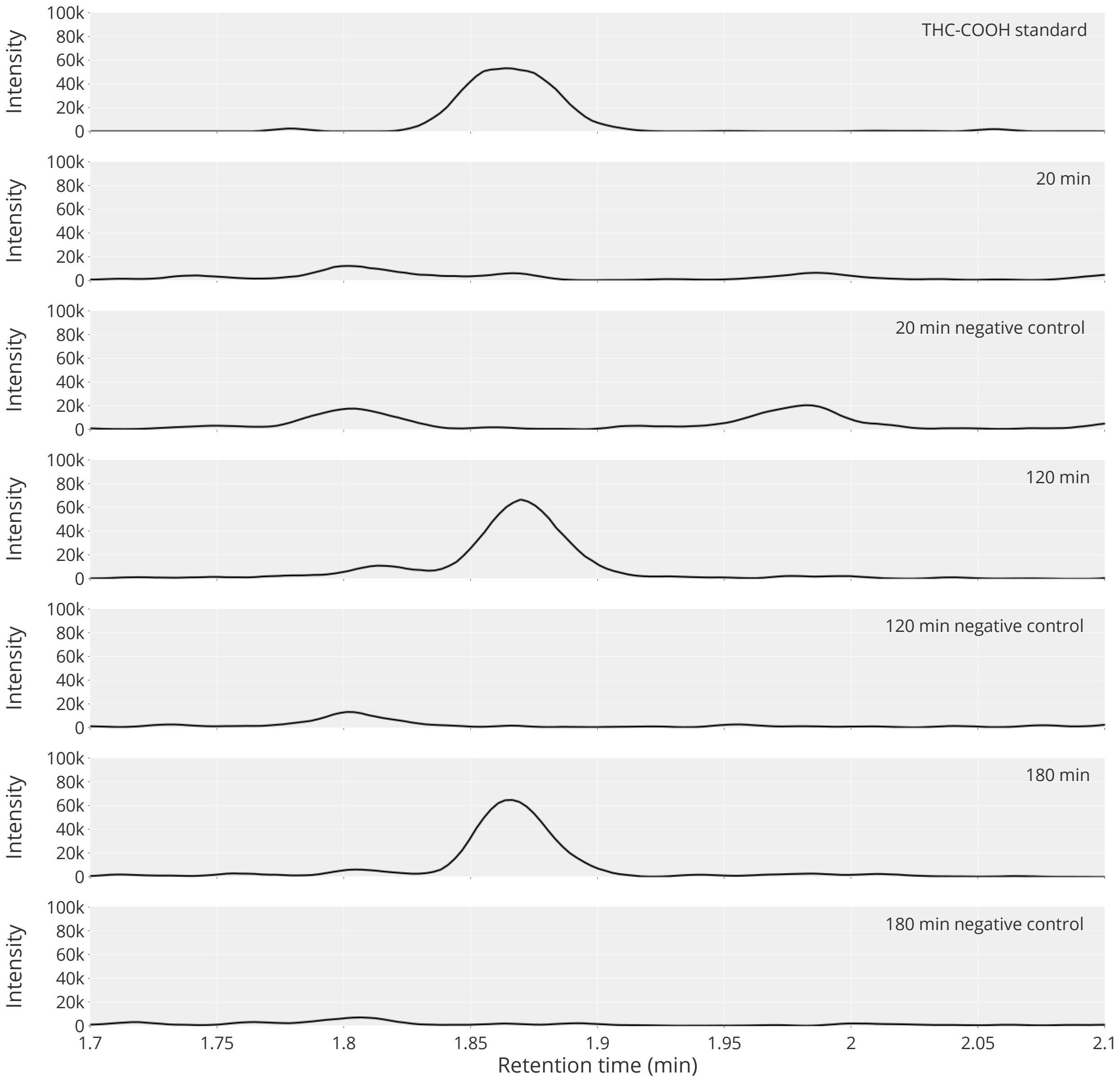

Figure S10. Chromatogram showing 11-nor-9-carboxy-tetrahydrocannabinol (THC-COOH) in incubation of 11-hydroxy-tetrahydrocannabinol (11-OH-THC) with pooled human liver microsomes for 20, 120 and 180 minutes. Negative controls are incubations without the NADPH regenerating system. Mass transition: 345>193.

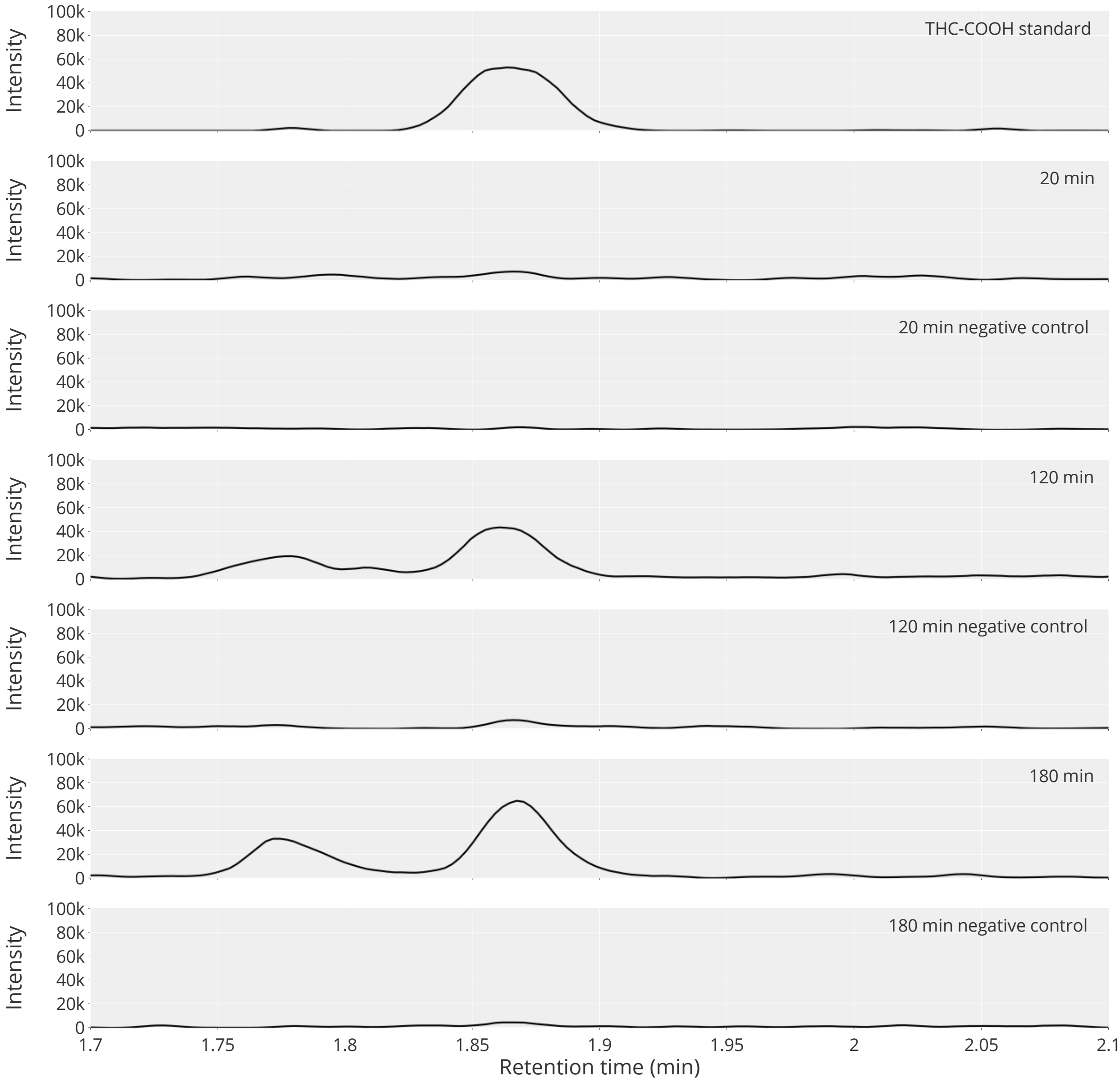

Figure S11. Chromatogram showing 9R- and 9S-epimers of 11-hydroxy-hexahydrocannabinol (11-OH-HHC) and 11-hydroxy-tetrahydrocannabinol (11-OH-THC) in a standard solution containing 11-OH-THC. Mass transitions: 331>193 (top) and 333>193 (bottom).

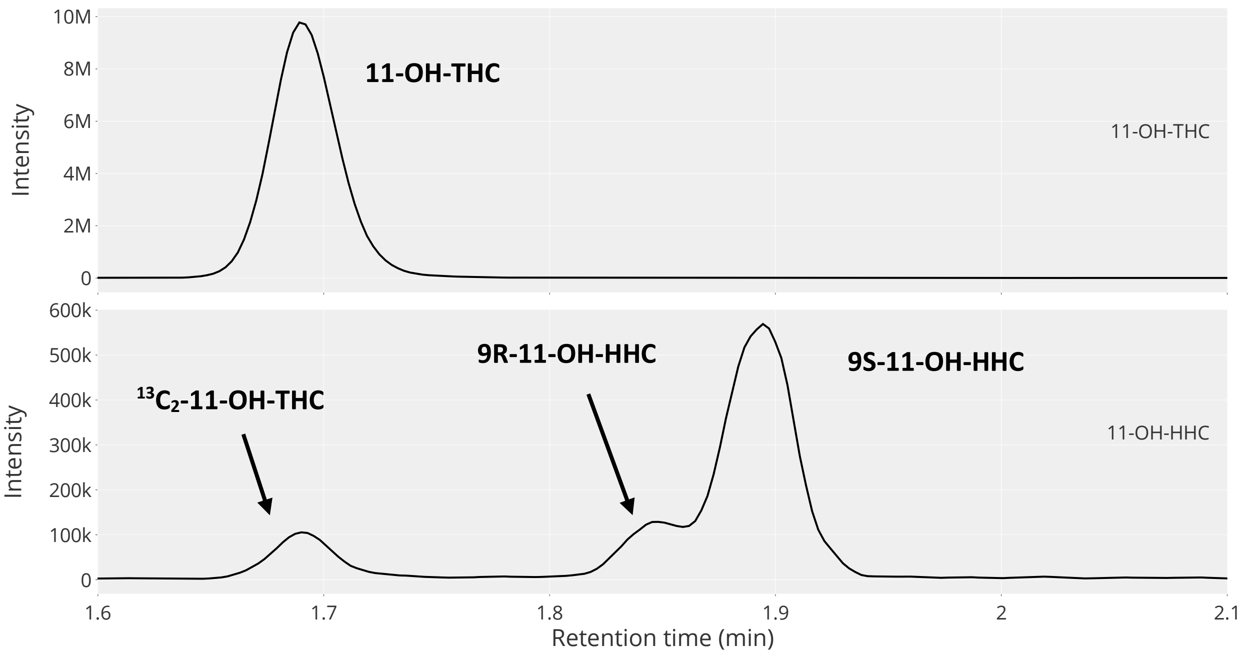

Supplement: Supplementary file 1 [file metabolites-13-01169-s001.zip › metabolites-2652779-supplementary.pdf]
